# Compound heterozygous variants of the *SLC26A4* gene in a Chinese family with enlarged vestibular aqueducts

**DOI:** 10.1186/s12920-022-01271-3

**Published:** 2022-07-08

**Authors:** Xiaohui He, Shaozhi Zhao, Lin Shi, Yitong Lu, Yintong Yang, Xinwen Zhang

**Affiliations:** Xi’an People’s Hospital (Xi’an Fourth Hospital), Xi’an, China

**Keywords:** Enlarged vestibular aqueducts, *SLC26A4*, A novel missense mutation, c.2069T>A

## Abstract

**Background:**

To investigate the genetic causes of hearing loss in patients with enlarged vestibular aqueduct (EVA), the *SLC26A4*-related genotypes and phenotypes were analyzed. *SLC26A4* gene is closely associated with EVA and its homozygous mutations or compound heterozygous mutations may cause deafness and strongly affect quality of life.

**Methods:**

The patients who came to our hospital for hearing test and accompanied by bilateral hearing abnormalities were collected for fifteen deafness-related gene mutations detection. Those who are positive will be verified by Sanger sequencing, combined with family history, hearing test, and computerized tomography (CT) of the temporal bone, aiming to diagnose the enlarged vestibular aqueducts. Whole-exome sequencing were performed when necessary.

**Results:**

Our patient failed hearing screening on both sides twice, and EVA (> 1.5 mm) was diagnosed by CT. This study has identified a novel missense mutation in the *SLC26A4* gene, c.2069T>A, which in compound heterozygosity with c.1174A>T is likely to be the cause of hearing loss. The novel heterozygous c.2069T>A mutation of *SLC26A4* gene has been submitted to Clinvar with Variation ID 1,048,780.

**Conclusion:**

Our findings expand the gene mutation spectrum of *SLC26A4* and provide additional knowledge for diagnosis and genetic counseling associated with EVA-induced hearing loss.

**Supplementary Information:**

The online version contains supplementary material available at 10.1186/s12920-022-01271-3.

## Introduction

As one of the most serious sensory defects, hearing loss not only affects the quality of life, but also affects physical and mental health [1]. Inner ear malformations, with enlarged vestibular aqueduct (EVA) as the most common one, are found in many patients with hearing abnormalities. In children, sensorineural hearing loss is also commonly associated with inner ear malformations [2]_._ EVA patients have non-syndromic hearing loss, and a small number of patients with goiter are called Pendred syndrome (PDS) [3]. The *PDS* gene that can cause Pendred syndrome (PDS) and non-syndromic deafness was later renamed *SLC26A4* and located on chromosome 7q [4]. *SLC26A4* has 21 exons and encodes the protein pendrin which expressed in thyroid, kidney and inner ear. Pendrin plays a role in the transport of anions between the thyroid and inner ear [5]. One phenotype of *SLC26A4* mutants is temporal bone developmental malformations, including large vestibular aqueduct syndrome (LVAS) and Mondini malformation.

Hot spot variants in the *SLC26A4* gene reflect regional and ethnic differences. For the Chinese mutation spectrum of *SLC26A4* gene, the c.919-2 A>G mutation and c.2168A>G mutation account for most of mutations in China. The c.707T>C, c.1246A>C and c.1001+ 1G>A mutations are mainly detected in Caucasians. The c.1826T>G and c.1001 + 1G>A mutations are quite common in South America and North America. The c.2168A>G mutation is mainly discovered in Koreans [6].

A total of 8647 mutations have been reported in *SLC26A4*, of which 487 are Pathogenic mutations, and likely pathogenic mutations are 118. (https://deafnessvariationdatabase.org/gene/SLC26A4). Missense mutations account for the majority, followed by frameshift mutations and splice site mutations. Exon 8 and its flanking sequences have been reported to be a highly variable region where multiple mutations located, followed by exon 19, 10, 17, and 15 [7].

Here, we report a case of sensorineural hearing loss that presented to our hospital. Through bilateral hearing test and CT imaging of the temporal bone, we made a preliminary diagnosis of sensorineural hearing loss. In order to further investigate genetic factors, the microarray method was applied to screen 15 common mutations in 4 deafness genes (*GJB2* c.35delG, c.176_191del, c.235delC, and c.299_300del AT; *GJB3* c.538C>T; *SLC26A4* c.1174 A>T, c.1226G>A, c.1229C>T, c.1975G>C, c.2027T>A, c.2168A>G, c.919-2A>G, c.1707 + 5G>A; MT-RNR1 m.1555A>G and m.1494C>T). The proband and her mother have a heterozygous mutation in the *SLC26A4* gene (c.1174A>T), while her father is wild-type at 15 loci. There was no abnormality in the hearing of the parents. Then, whole-exome sequencing and Sanger sequencing were performed to uncover the genetic cause of proband’s hearing loss. A novel heterozygous mutation in *SLC26A4* gene was identified.

## Proband and method

### Clinical symptoms of the proband

The proband was a 3 years old girl with hearing loss in both ears, symptoms recurring with occasional tinnitus. Based on physical examination, our patient’s double external auditory canals were unobstructed, there was no viscous secretion, and the tympanic membrane was normal. The history of surgical trauma, hypertension, diabetes, and heart disease were denied.

Our patient failed the first hearing screening, and the second hearing screening (December 11, 2018) failed in the left ear but passed in the right ear. However our patient failed in both ears again on April 2, 2019 and was diagnosed with EVA by CT on May 14, 2019. The imaging findings including: (1) Inner ear: The bilateral internal auditory canals were symmetrical without enlargement and stenosis, with full bilateral vestibules and enlarged vestibular aqueduct. The right side was about 4.1 mm wide, and the left side was about 4.4 mm wide. The spiral tubes of the bilateral cochlea appeared to be a circle and a half, with clear semicircular canal structure and normal shape. There was no high position of the bilateral jugular bulbs. (2) Middle ear: The bilateral mastoid processes were well developed, with gasification shape and no abnormal density. The mastoid sinus and tympanic sinus showed no abnormal density shadow. There was no enlargement of the tympanum, and the structure of the ossicles was clear without damage. (3) The outer ear canal was unobstructed, and small flake-like high-density shadows can be seen in it. The diagnosis was bilateral vestibular aqueducts dilatation, with the exception for cochlear spiral duct malformations. The hearing of her parents was normal.

### Genetic testing methods

After the patient and her parents have signed the informed consent document, 15 genetic tests for hereditary deafness were conducted. To further find out the causes, the patient accepted the whole-exome sequencing and Sanger sequencing was used to verify her parents.

#### Fifteen deafness-related gene mutations test

This method mainly used a test kit. Human genomic DNA was used as a template, loci-specific primers with tag sequences were used to amplify and label the relevant gene fragments with fluorescence and biotin. After magnetic separation and Alkaline denaturation were applied, the DNA fragments were hybridized with the cloned sequences on a universal gene chip that capable of recognizing the corresponding tag. Finally, test results of the 15 tested loci were obtained by scanning the chip and data analyzing. Since the primers and probes were designed for both wild-type and mutant type of the 15 tested loci, the wild-type and mutant-type were tested simultaneously.

#### Whole-exome sequencing analysis

The genomic DNA was extracted, hybridized and enriched. Novaseq6000 platform (Illumina, San Diego, USA) was used for sequencing the genomic DNA of our patient. Raw image files were processed using CASAVA v1.82 for base calling and generating raw data.Verita Trekker® Variants Detection System Genomics and the third-party software GATK were employed for variant calling. Variant annotation and interpretation were conducted by ANNOVAR and the Enliven® Variants Annotation Interpretation System. The analysis filtered out variants with a mutation frequency greater than 1% in the Human Exon Database (ExAC), the 1000 Genomes Project, and the Genome Aggregation Database (gnomAD). Non-functional variant sites were then filtered. Pathogenicity prediction was performed using a variety of software including SIFT, MutationAssessor, Polyphen2, CADD and others. Analysis was carried out through disease and phenotype databases, including HGMD, OMIM, ClinVar, and others. Finally, potential pathogenic variants were obtained.

#### Sanger sequencing and family analysis

The pathogenic variant was detected in the proband by WES and Sanger sequencing. Then Sanger sequencing validation was used for family analysis. The Sanger sequencing was performed on the ABI 3500DX. Pathogenicity classification of genetic variants was based on American Association for Medical Genetics and Genomics (ACMG) guidelines [8].

## Results

### Results of genetic testing

**Fig. 1:**
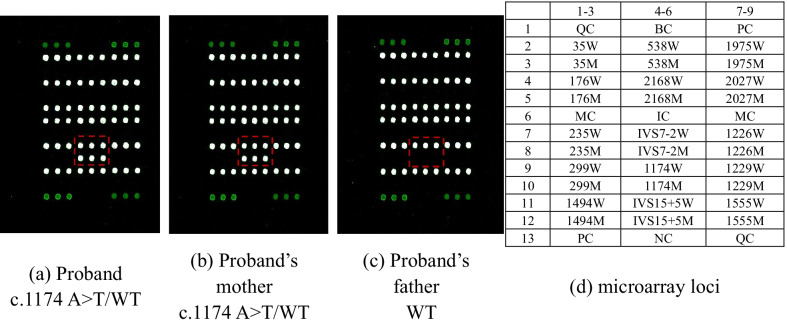
The results of fifteen Deafness-Related Gene Mutations Detection Kit (Microarray)

**Fig. 2:**
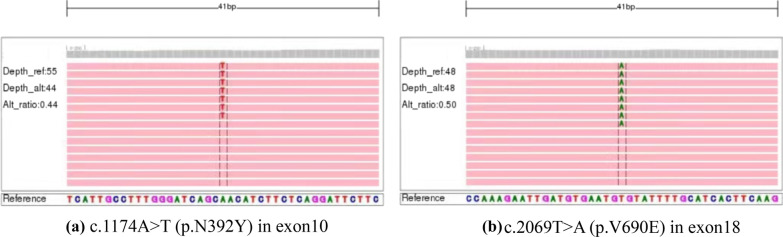
Compound heterozygous mutations (N392Y and V690E) of *SLC26A4* gene in the proband identified by whole-exome sequencing (Integrative Genomics Viewer)

**Fig. 3:**
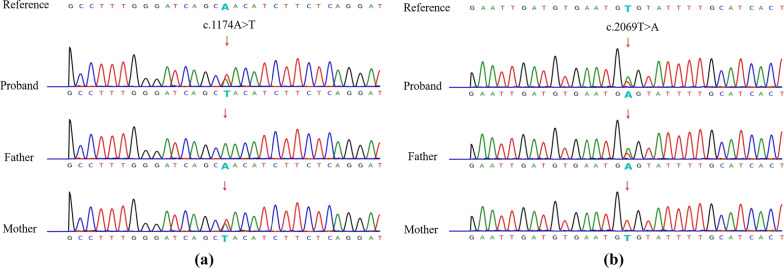
Sanger sequencing confirmed the mutation of the proband and verified her parents

Our patient and parents accepted fifteen deafness-related gene mutations test, and the results are shown in Fig. 1 and Additional file 1. Our proband and her mother have a heterozygous mutation in the *SLC26A4* gene (c.1174A > T), and her father is wild-type (WT) at 15 loci. As our patient’s mother was pregnant, in order to figure out the causes of the patient’s hearing abnormalities and provide genetic counseling, whole-exome sequencing was performed in the proband and Sanger sequencing was used to test her parents. The results of whole-exome sequencing analysis are displayed in Fig. 2 and only mutated base is highlighted. As shown in Fig. 2a, the c.1174 of *SLC26A4* from proband is T while the reference sequence is A. Depth_rel:55 means that there are 55 reads same as the reference base, and Depth_alt:44 means that 44 reads are T. Alt_ratio: 0.44 means the mutation ratio is 0.44 (44%). Figure 2b indicates the sequencing result at this position is A and the reference sequence is T. Depth_rel:48 means that there are 48 reads same as the reference base, and Depth_alt:48 means that 48 reads are altered from reference. Alt_ratio: 0.50 means the mutation ratio is 0.50 (50%). The results of Sanger sequencing are listed in Table 1 and Fig. 3. The proband carries compound heterozygous variants of c.1174A>T (p.N392Y) and c.2069T>A (p.V690E) of *SLC26A4*. Her mother has heterozygous mutation of c.1174A>T in the *SLC26A4* gene. Her father carries heterozygous mutation of c.2069T>A in the *SLC26A4* gene.

**Table 1:** The results of Sanger sequencing

Gene	Mutation location	Gene subregion	HGVS	Heterozygosity
*SLC26A4*	chr7:107,690,148	exon10	NM_000441.2:c.1174 A > T:p.N392Y	Proband: Heterozygous Mother: Heterozygous Father: wild
*SLC26A4*	chr7:107,704,365	exon18	NM_000441.2:c.2069 T > A:p.V690E	Proband: Heterozygous Father: Heterozygous Mother: wild

### The pathogenicity rating of the variants

According to American College of Medical Genetics and Genomics (ACMG) guidelines, the c.1174A>T (p.N392Y) mutation in the *SLC26A4* was classified as pathogenic by fulfilling the standard PM3_VeryStrong, PM1, PP3, PP4, PS3_Supporting and PM2_Supporting.

Based on the literature, the pathogenic or likely pathogenic variants were detected translocations of variants in 3 individuals with deafness [9] (PM3_VeryStrong). The variant located in the functional structure of *SLC26A*/SulP transporter domain (PM1). The predicted results by multiple statistical methods (REVEL) revealed that the mutation has harmful effects on genes or gene products (PP3). The corresponding disease of the variant is consistent with the phenotype of this case (PP4). It has been reported that this mutation caused gene function damage in immunofluorescence experiments [10] (PS3_Supporting). This mutation is collected by the China Genome Database (0.00071531), the Human Exome Database (ExAC) (8.24198466990851e-06), the reference population Thousand Genome (1000G) (0.000199681) and the Population Genome Mutation Frequency Database (gnomAD) (0.00019253). This known variant is assessed as pathogenic in the ClinVar database and DM in the HGMD database [11–14] (PM2_Supporting), respectively.

According to the ACMG guidelines, the c.2069T>A (p.V690E) of *SLC26A4* gene is classified as likely pathogenic by fulfilling the criteria PM1, PM2, PM3, PP3 and PP4.

The mutation c.2069T>A of *SLC26A4* affects the functional STAS domain (PM1). The mutation in the China Genome Database, the Human Exome Database (ExAC), the Reference Population Thousand Genome (1000G) and the Population Genome Mutation Frequency Database (gnomAD) is not found (PM2). This variant forms a compound heterozygosity with the c.1174A>T mutation site in the *SLC26A4* gene (PM3). The predicted results by a variety of statistical methods (REVEL), indicate that the variant encoded genes or gene products cause harmful effect (PP3). The corresponding disease of the variant matches the EVA phenotype of this case (PP4).

## Discussion and conclusion

It was found that our patient had the c.1174A > T (p.N392Y) and c.2069T>A (p.V690E) mutations of *SLC26A4*. The c.2069T>A mutation of *SLC26A4* has not been reported or categorized, which leads to a compound heterozygosity with the c.1174A>T mutation in our patient. The mutation affects an amino acid in the STAS domain (amino acid position 535–729) which is functional and highly conserved. The variant may affect the protein function of SLC26A4. Another missense mutation at the same position was found in a patient with EVA, p.V690A, but results in a different amino acid change. There are no functional analyses or other reports on the p.V690A mutation of *SLC26A4* gene. It has been reported another compound heterozygosity of the *SLC26A4* which has the c.1341 + 1G>C mutation and the c.2069T>C mutation in one patient with EVA [15]. The HGMD database rates the c.1341 + 1G>C mutation and the c.2069T>C mutations of *SLC26A4* as DM. Therefore, it does not support the c.2069T>A mutation of *SLC26A4* as the evidence PM5.

**Fig. 4:**
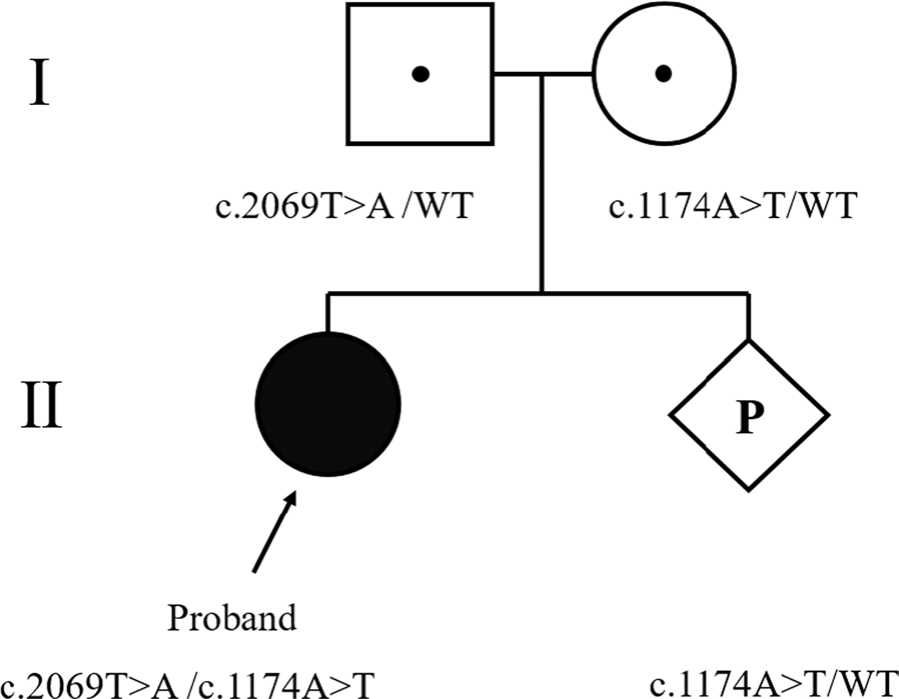
*SLC26A4* mutations in the family with EVA [16]

Elucidating the influences of *SLC26A4* gene function has a certain guiding role in the later cochlear implantation or other treatments for deaf patients. Moreover, it has become an important supplementary method for temporal bone CT screening, and *SLC26A4* gene mutations testing can detect and diagnose LVAS during neonatal hearing screening. Our patient’s mother was pregnant again and her fetus was heterozygous mutation of c.1174A>T in the *SLC26A4* gene (Fig. 4). Genetic counseling recommended to continuing pregnancy. During a follow-up, the four-months-old baby girl, had a normal routine physical examination and passed the newborn hearing screening. It also clarified the goal for the follow-up prenatal diagnosis. Enriching the mutation spectrum of the *SLC26A4* gene has a complementary effect on the rare mutation sites of deafness. High-throughput sequencing technology has significant advantages in identifying the genetic factors of diseases. At present, high-throughput sequencing of fetal free DNA in maternal peripheral blood has opened a new direction for non-invasive prenatal diagnosis of hereditary deafness, which will further promote genetic counseling.

As a membrane transport protein exchanging anions between the cytoplasm and extracellular fluid, pendrin can mediate chloride (Cl^−^), hydroxide (OH^−^), bicarbonate (HCO3^−^) and iodide (I^−^) exchanging [17]. Studies have illustrated the role of *KCNJ10* and *FOXI1* in PDS, and it has been suggested that double gene mutations, namely *SLC26A4* and *FOXI1* or *KCNJ10,* may cause PDS [18]. FOXI1 can bind SLC26A4 and mediate its activation and transcription [19]. However, the correlation between *KCNJ10, FOXI1* and PDS is proven to be extremely weak in other studies. It is very unlikely that these genes will be screened in patients with EVA. Other factors such as genetics or environment may play a more important role in the etiology of PDS/EVA [20–22].

According to some studies, the transport function of SLC26A4 protein can be examined through cell experiments, so that the activity of SLC26A4 protein can be predicted [23]. In previous transfected HEK293 cells study, some disease related *SLC26A4* mutants affect the transport process of pendrin rather than its expression level [10]. However, the study also discussed that reduced membrane expression and trafficking activity of mutant pendrins was the pathogenesis of hearing loss in patients with EVA [24]. The human gene *SLC26A4* is homologous to the mouse *Slc26a4*, which encodes the protein. The studies on this mouse model revealed that the pathophysiological mechanisms are related to the loss of function or hypofunction of *SLC26A4* [25, 26]. Some reports established transgenic mice for *Slc26a4* variants to mimic its pathogenic process [27]. The identity of SLC26A4 between mice and humans is only 86%. The amino acid identity of the transmembrane domain is 92%, but the C-terminus of pendrin is less conserved. Therefore, it was speculated that the lacking phenotype of the *Slc26a4* C-terminal variant mouse could be ascribed to the different protein structures of C-terminus. (https://www.uniprot.org/). The p.V690E variant is also C-terminal mutation and may not benefit from making a mouse model. We plan to focus on the function of p.V690E, using HEK293 cells transfected with a plasmid containing p.V690E mutation of the *SLC26A4* gene to analyze its cellular localization and anion exchange activity, to verify the pathogenicity of this site through a series of functional tests.

This study reports that the presence of a novel missense mutation c.2069T>A in the *SLC26A4* gene with c.1174A>T leading to compound heterozygosity as the cause of deafness. Our findings will expand *SLC26A4* gene mutation spectrum and provide additional information for diagnosis and genetic counseling that is associated with EVA-induced hearing loss.

## Supplementary Information

**Additional file1**: **Table S1.** Details explanation of fifteen deafnessrelated mutation loci.

## Data Availability

The details of the variant analyzed during the current study are available in the ClinVar repository, under the Accession Number SCV001548357.1. (https://www.ncbi.nlm.nih.gov/clinvar/variation/1048780/?new_evidence=false) The raw datasets generated during the current study are not publicly available because it is possible that individual privacy could be compromised. It is possible to apply for permission to obtain access to the raw sequencing data through the corresponding author.

## Abbreviations

CT: Computerized tomography
EVA: Enlarged vestibular aqueduct
LVAS: Large vestibular aqueduct syndrome
ACMG: American College of Medical Genetics and Genomics guidelines
PDS: Pendred syndrome
