# Supplementary material for: Compound heterozygous variants of the SLC26A4 gene in a Chinese family with enlarged vestibular aqueducts
Source: BMC Med Genomics. 2022 Jul 8;15:152. doi: 10.1186/s12920-022-01271-3 (PMC9270741; doi:10.1186/s12920-022-01271-3)
Supplement: Supplementary file 1 — Additional file1: Table S1. Details explanation of fifteen deafnessrelated mutation loci. [file 12920_2022_1271_MOESM1_ESM.docx]

**[Supplementary Materials](https://www.ncbi.nlm.nih.gov/pmc/articles/PMC4375444/)**

Supplementary Table 1

| Kind of probe | Probe name | Probe Meaning | Probe name | Probe Meaning |
| --- | --- | --- | --- | --- |
| Quality control probe | QC | Surface Chemistry Quality Control | BC | Blank control |
|  | PC | Hybrid positive control | NC | Negative control |
|  | IC | Gene Amplification Internal Control | MC | Magnetic Bead Quality Control |
| gene locus detection probe | 35W | wild-type | 35M | *GJB2*: c. 35delG |
|  | 176W | wild-type | 176M | *GJB2*: c. 176_191del |
|  | 235W | wild-type | 235M | *GJB2*: c. 235delC |
|  | 299W | wild-type | 299M | *GJB2*: c. 299_300del AT |
|  | 538W | wild-type | 538M | *GJB3*: c. 538 C>T |
|  | 1494W | wild-type | 1494M | MT-RNR1 m.1494 C > T |
|  | 1555W | wild-type | 1555M | MT-RNR1 m.1555A>G |
|  | 1174W | wild-type | 1174M | *SLC26A4*: c. 1174 A>T |
|  | 1226W | wild-type | 1226M | *SLC26A4*: c. 1226 G > A |
|  | 1229W | wild-type | 1229M | *SLC26A4*: c. 1229 C > T |
|  | 1975W | wild-type | 1975M | *SLC26A4*: c. 1975 G > C |
|  | 2027W | wild-type | 2027M | *SLC26A4*: c. 2027 T > A |
|  | 2168W | wild-type | 2168M | *SLC26A4*: c. 2168 A > G |
|  | IVS7-2W | wild-type | IVS7-2M | *SLC26A4*: c. 919-2 A > G |
|  | IVS15+5W | wild-type | IVS15+5M | *SLC26A4*: c. 1707+5 G > A |
